# Repeated adrenocorticotropic hormone administration alters adrenal and thyroid hormones in free-ranging elephant seals

**DOI:** 10.1093/conphys/coy040

**Published:** 2018-07-17

**Authors:** Molly C McCormley, Cory D Champagne, Jared S Deyarmin, Alicia P Stephan, Daniel E Crocker, Dorian S Houser, Jane I Khudyakov

**Affiliations:** 1 Department of Biological Sciences, University of the Pacific, Stockton, CA, USA; 2 Conservation and Biological Research Program, National Marine Mammal Foundation, San Diego, CA, USA; 3 Biology Department, Sonoma State University, Rohnert Park, CA, USA

**Keywords:** Aldosterone, cortisol, ACTH, HPA axis, marine mammals, stress, thyroid

## Abstract

Understanding the physiological response of marine mammals to anthropogenic stressors can inform marine ecosystem conservation strategies. Stress stimulates the activation of the hypothalamic–pituitary–adrenal (HPA) axis and synthesis of glucocorticoid (GC) hormones, which increase energy substrate availability while suppressing energy-intensive processes. Exposure to repeated stressors can potentially affect an animal’s ability to respond to and recover from subsequent challenges. To mimic repeated activation of the HPA axis by environmental stressors (or challenges), we administered adrenocorticotropic hormone (ACTH) to free-ranging juvenile northern elephant seals (*Mirounga angustirostris*; *n* = 7) once daily for 4 days. ACTH administration induced significant elevation in circulating cortisol and aldosterone levels. The cortisol responses did not vary in magnitude between the first ACTH administration on Day 1 and the last administration on Day 4. In contrast, aldosterone levels remained elevated above baseline for at least 24 h after each ACTH injection, and responses were greater on Day 4 than Day 1. Total triiodothyronine (tT3) levels were decreased on Day 4 relative to Day 1, while reverse triiodothyronine (rT3) concentrations increased relative to baseline on Days 1 and 4 in response to ACTH, indicating a suppression of thyroid hormone production. There was no effect of ACTH on the sex steroid dehydroepiandrosterone. These data suggest that elephant seals are able to mount adrenal responses to multiple ACTH administrations. However, repeated ACTH administration resulted in facilitation of aldosterone secretion and suppression of tT3, which may impact osmoregulation and metabolism, respectively. We propose that aldosterone and tT3 are informative additional indicators of repeated stress in marine mammals.

## Teaser

The ability to identify stressed marine mammals is essential for understanding physiological consequences of disturbance. We simulated repeated stress in elephant seals by repeated administration of adrenocorticotropic hormone and examined their resulting endocrine responses. Altered aldosterone and thyroid hormone levels distinguished acute from repeated responses to adrenocorticotropic hormone administration in seals.

## Introduction

Anthropogenic disturbance can impact vulnerable wildlife populations by compounding allostatic loads experienced by individuals as a result of natural challenges (Romero *et al.*, 2009). Chronic stress, which can be caused by sustained or repeated challenges, can lead to homeostatic overload, impairing an animal’s ability to respond appropriately to additional challenges and potentially impacting survival (Rich and Romero, 2005; Romero *et al.*, 2009). Changes in the health and abundance of wildlife populations, especially of top predators like marine mammals, can have ecosystem-wide consequences (Maxwell *et al.*, 2013). Marine mammals routinely experience physiological challenges such as hypoxia and prolonged fasting, and amphibious species (e.g. pinnipeds, or seals and sea lions) are exposed to both terrestrial and aquatic stressors (Ridgway, 1972; Ponganis, 2011). Increased anthropogenic activity in coastal and marine habitats, such as noise pollution, commercial fishing and human-driven habitat loss, in addition to natural environmental challenges (e.g. prey availability), are correlated with modern declines in marine mammal populations (Springer *et al.*, 2003; Atkinson *et al.*, 2008; Maxwell *et al.*, 2013). Most recent research on stress in marine mammals has focused on animals’ responses to acute stressors (e.g. a single endocrine response; Mashburn and Atkinson, 2008; Ensminger *et al.*, 2014; Champagne *et al.*, 2015; Keogh and Atkinson, 2015; Khudyakov *et al.*, 2017). However, little information exists on impacts and indicators of chronic stress (e.g. endocrine response to repeated or sustained stressors) in free-ranging marine mammals and most other wildlife species (Fair and Becker, 2000; Dickens and Romero, 2013). Therefore, evaluation of the physiological impacts of both acute and chronic stress in marine mammals is necessary to understand how populations may respond to anthropogenic and environmental disturbance over time and can help inform conservation management strategies (Cooke *et al.*, 2010; Dantzer *et al.*, 2014; Jessop *et al.*, 2013).

The mammalian stress response is mediated by the autonomic nervous system and the hypothalamic–pituitary–adrenal (HPA) axis (Sapolsky *et al.*, 2000; Atkinson *et al.*, 2015). Activation of the HPA axis by an acute stressor induces a series of cascading events, causing the synthesis of glucocorticoids (GC), mineralocorticoids and androgens by the adrenal gland as a result of stimulation by adrenocorticotropic hormone (ACTH) released from the pituitary gland, which is induced by corticotropin-releasing hormone (CRH) released from the hypothalamus (Sapolsky *et al.*, 2000). GCs (e.g. cortisol) are considered the primary stress hormones; they exert their effects by binding to membrane (nongenomic) and intracellular (genomic) glucocorticoid receptors and mineralocorticoid receptors (MR) in target tissues and influencing signalling pathways and gene expression (Sapolsky *et al.*, 2000; de Kloet *et al.*, 2008; Lösel and Wehling, 2008). GCs exert numerous physiological effects, which include increased cognitive activity and emotional arousal, catabolism of metabolic stores required to meet immediate energetic needs and suppression of energetically-demanding functions (e.g. reproduction and immune function; Dhabhar, 2002; Romero, 2004; Crossin *et al.*, 2016). Negative feedback at the receptor level in the hypothalamus, pituitary and paraventricular nucleus is responsible for regulating synthesis of GCs to avoid potentially deleterious long-term effects of their sustained elevation, such as depletion of energy reserves needed for organismal maintenance and reproduction. Such effects can have especially detrimental consequences for marine mammal species that rely on stored energy reserves (i.e. blubber) to sustain prolonged fasting periods associated with reproduction, molt or migration. While many studies of stress in wild animals have concentrated solely on GC measurements, no consensus GC profile has been determined that characterizes sustained or repeated stress responses (Dickens and Romero, 2013). Therefore, a suite of additional hormones should be measured to fully evaluate the consequences of repeated stress (Rushen, 1986). These include other adrenal hormones (aldosterone and dehydroepiandrosterone, DHEA) and components of the hypothalamic–pituitary–thyroid (HPT) axis.

The mineralocorticoid aldosterone is a primarily osmoregulatory hormone that has not been studied extensively in the context of the stress response in terrestrial mammals (Kubzansky and Adler, 2010). In marine mammals, however, a number of studies have demonstrated significant increases in aldosterone secretion in response to perturbation (Gulland *et al.*, 1999; Ensminger *et al.*, 2014; Champagne *et al.*, 2015; Khudyakov *et al.*, 2015, 2017; Burgess *et al.*, 2017), suggesting that mineralocorticoids may be an especially important component of the stress response in mammals adapted to hypersaline environments, with potential osmoregulatory or cardiovascular costs (Milliez *et al.*, 2005; Ortiz *et al.*, 2006; Garg and Adler, 2008; Kubzansky and Adler, 2010; Ponganis, 2011). The sex steroid precursor DHEA (commonly measured in its more abundant sulfated form, DHEA-S) is produced by the adrenal cortex in response to HPA axis activation in vertebrates using similar precursors and biosynthetic pathways to GCs (Boonstra *et al.*, 2008; Newman *et al.*, 2008; Newman and Soma, 2009; Lennartsson *et al.*, 2012). Repeated stress, therefore, may influence the synthesis of DHEA (via GC effects on expression of the enzymes 17α-hydroxylase, CYP17A1 and 3β-hydroxysteroid dehydrogenase, 3βHSD), which has been shown to counteract GC activity and alter lipid metabolism, insulin sensitivity and adipocytokine production in adipose tissue (Morgan *et al.*, 2004; McNelis *et al.*, 2013). Dysregulation of DHEA during repeated stress may thus impact reproduction and metabolism. HPA axis activation also directly affects the HPT axis by decreasing thyroid stimulating hormone release and inhibiting biologically active triiodothyronine (T3) synthesis, leading to an increase in the inactive isomer reverse triiodothyronine (rT3; Biebuyck *et al.*, 1990; Charmandari *et al.*, 2005). Thyroid hormones, which regulate basal metabolic rate, thermogenesis and lipid metabolism, have been shown to be important for fasting metabolism in elephant seals (Crocker *et al.*, 2012; Jelincic *et al.*, 2017). Suppression of the thyroid axis by repeated stress would have significant detrimental consequences on the ability of marine mammals to effectively undertake energetically-demanding key life-history stages that require fasting.

The objective of this study was to characterize the endocrine responses to acute and repeated ACTH administration in a well-studied marine mammal, the northern elephant seal (*Mirounga angustirotris*), and evaluate their potential for discrimination between these states. We simulated acute and repeated stress responses by daily administration of synthetic ACTH for four consecutive days and compared responses to initial and subsequent ACTH administrations. Due to their accessibility on land and relative ease of research handling, considerable amount of data are available on baseline physiological variables in elephant seals, including natural variability in GCs, thyroid and other hormones (Ortiz *et al.*, 2001; Fowler *et al.*, 2008; Crocker *et al.*, 2012; Jelincic *et al.*, 2017). Furthermore, sedation procedures are well-established for this species, enabling experimental manipulation and sample collection without the artefact of handling stress (Champagne *et al.*, 2012). Here, we describe endocrine responses to a repeated ACTH administration experiment in a free-ranging marine mammal, which include significant alterations in corticosteroids and thyroid hormones that varied with acute and repeated ACTH responses.

## Methods

### Experimental design

**Figure 1:**
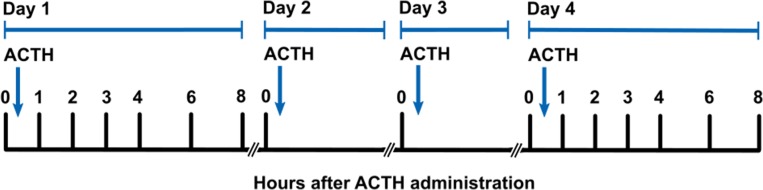
Overview of the study design, timing of ACTH administration, and blood sample collection during the 4-day experiment. ACTH was administered once daily. Blood samples were collected immediately prior to ACTH administration (0-h) on each day. Post-ACTH samples were collected on Days 1 and 4 only, 1–4, 6, and 8 h following the administration.

The ACTH administration experiment was conducted August–October 2016 at Año Nuevo State Park, San Mateo County, CA, USA. Experiments were conducted using seven juvenile northern elephant seals. ACTH was administered once daily for four consecutive days to simulate an acute and repeated stress response. Serial blood samples were collected pre-ACTH (0-h) and post-ACTH (1–8 h) on Days 1 and 4 assess the effects of ACTH administration on cortisol, aldosterone and thyroid hormones (Fig. 1).

### Study subjects

All animal handling procedures were conducted under National Marine Fisheries Service permit 19108 and approved by Sonoma State University and University of the Pacific Institutional Animal Care and Use Committees and Department of the Navy Bureau of Medicine and Surgery. Juvenile northern elephant seals regularly haul out during fall and are reliably found on successive days at the rookery. Natural fluctuations in corticosteroid levels in response to life-history stages, such as molting and breeding, are not associated with this haul out period (Kelso *et al.*, 2012; Jelincic *et al.*, 2017). The study subjects, two females and five males, were qualitatively selected based on age (~0.8 year-old), apparent mass and body condition (Table 1). All study animals appeared healthy and were within the normal mass range for their age (99–137 kg; Table 1). Seals were weighed by suspension from a tripod and scale (MSI tension dynamometer, Seattle, WA, USA), and marked with rear flipper tags (Dalton, Oxon, UK) for identification. All study animals resumed normal activity and remained at the rookery after the experiment was completed each day.

**Table 1:** Serum cortisol (Cort) and aldosterone (Aldo) concentrations measured on Days 1 and 4 of the repeated ACTH administration experiment in seven juvenile elephant seals. Baseline (Base) samples were taken before ACTH administration. Peak is the highest concentration measured after ACTH administration within the 8-h sampling period. Sex, mass and mass-specific ACTH dose (U/kg) for each subject are shown

				Day 1				Day 4			
Subject	Sex	Mass (kg)	ACTH (U/kg)	Base Cort (nM)	Peak Cort (nM)	Base Aldo (pM)	Peak Aldo (pM)	Base Cort (nM)	Peak Cort (nM)	Base Aldo (pM)	Peak Aldo (pM)
1	M	125	0.16	55	1199	1478	1861	593	2641	1447	2155
2	M	119	0.19	199	1066	813	1466	202	2626	1204	2111
3	M	130	0.15	151	1337	753	1189	454	1323	821	1184
4	M	125	0.16	429	1703	828	2149	228	1924	1477	2609
5	M	137	0.15	444	1719	1058	2312	143	1436	1208	3044
6	F	103	0.19	358	1936	671	1251	494	4497	1610	2891
7	F	99	0.20	394	2599	792	1570	356	2319	1291	2242
**Mean (SD)**		**120 (13)**	**0.17 (0.02)**	**290 (142)**	**1651 (483)**	**913 (255)**	**1685 (403)**	**359 (156)**	**2395 (988)**	1294* (234)	2319* (573)

*Denotes statistically significant difference in mean peak and baseline hormone concentrations between Days 1 and 4. ACTH, adrenocorticotropic hormone.

### Sedation and ACTH administration

Seals were chemically immobilized using an intramuscular injection of ~1 mg/kg tiletamine–zolazepam HCl (Telazol, Fort Dodge Animal Health, Fort Dodge, IA, USA), and sedation was maintained with intravenous doses of ketamine and diazepam (Fort Dodge Animal Health, Fort Dodge, IA, USA) as needed to complete baseline sample collection and ACTH administration. This anaesthetic procedure does not affect GC concentrations (Champagne *et al.*, 2012). On each day of the experiment, pre-ACTH (0-h) samples were obtained from the extradural vein using an 18 G, 3.25-inch spinal needle within 18.0 ± 5.5 min of initial sedation. Samples were drawn directly into chilled vacuum collection tubes (serum, heparinized and EDTA-treated vacutainers; BD Franklin Lakes, NJ, USA).

Twenty units (U; mean mass-specific dose: 0.17 ± 0.02 U/kg) of a synthetic ACTH preparation (Khudyakov *et al.*, 2015, 2017; Wedgewood Pharmacy, Swedesboro, NJ, USA) were administered via intramuscular injection into the posterior flank of each animal following immobilization once daily for 4 days, 24.0 ± 0.7 h apart (Fig. 1). Alternate injection sites (e.g. Day 1: left side, Day 2: right side) were used each day. On Days 2 and 3, animals were allowed to recover from anaesthesia immediately after ACTH administration and no response samples were taken. On Days 1 and 4, samples were collected for 8 h after ACTH administration as described below.

### Post-ACTH sampling

On Days 1 and 4, an indwelling catheter (16 G × 20 cm, MILA International Inc, Florence, KY, USA) was inserted into the extradural vessel and attached to a 60-inch extension tube (MILA International Inc., Florence, KY, USA) after ACTH administration, following which the animals were allowed to recover from anaesthesia. Additional doses of ketamine and diazepam were administered prior to blubber tissue biopsy sampling for a related study on Days 1 and 4 as described previously (Khudyakov *et al.*, 2017). Serial blood samples, 1, 2, 3, 4, 6 and 8 h after ACTH administration were collected via extension tubing. Blood samples were drawn into syringes and immediately transferred to chilled vacuum collection tubes.

### Hormone analyses

Blood samples were stored chilled in a field cooler for 1–3 h until processing. Serum and plasma were isolated by centrifugation at 3000 × *g* for 15 min, kept frozen on dry ice until return to the laboratory, and stored at −80°C until further analysis.

Serum cortisol was measured in duplicate using a radioimmunoassay (RIA; catalogue # 06B256440, lot # FS1602, MP Biomedicals, Burlingame, CA, USA) previously validated for northern elephant seals (Khudyakov *et al.*, 2017). Aldosterone was measured in all plasma samples in triplicate using an enzyme-linked immunosorbent assay (ELISA; catalogue # 11-ALDHU-E01, lot # 161410; Alpco, Salem, NH, USA). To validate the aldosterone ELISA, we used a pool of elephant seal serum (with high concentrations of endogenous aldosterone collected during this study) to generate a series of serially diluted serum samples, which was parallel with the standard curve from the assay kit (Fig. S1A). We assessed parallelism from the linear region of the standard curve using an ANCOVA of the absorbance values against aldosterone concentration and sample type (kit standard or diluted serum); full model ANCOVA: *F*_1, 5_ = 643, *P* < 0.0001. The interaction term (aldosterone concentration * sample type) was not significant (*P* = 0.1), suggesting that the slopes do not significantly differ between the standards and serially diluted samples. Using a second pool of seal serum, we assessed the accuracy of the assay by evaluating observed values against those expected based on the degree of serum dilution. There was a significant relationship between the observed and expected values (observed = 506 + 0.79*expected; *r*^2^ = 0.98, *F*_1, 3_ = 197, *P* < 0.001; Supplementary Fig. S1B). The slope was within the accepted degree of accuracy (0.7–1.3; Grotjan and Keel, 1996). We tested for matrix interference effects by adding varying volumes (1–20 μL) of pooled seal serum (pooled from six samples) to a known volume of kit standard containing 425 μg aldosterone; total aldosterone concentrations were corrected for added standard (Beaulieu-McCoy *et al.*, 2017). There was no association between the corrected aldosterone concentration and volume of elephant seal serum added (*P* = 0.11). The calculated hormone recovery was 104 ± 6%. Average intra-assay coefficients of variation (CV) between sample replicates were 2.6% for cortisol and 5.9% for aldosterone. The inter-assay CV for aldosterone was 4.2%.

Thyroid hormones (total T3, tT3 and rT3) and DHEA-S were measured in a subset of serum samples (0-h samples from Days 1–4; 4-h and 8-h samples from Days 1 and 4) in duplicate using RIAs (tT3: catalogue # T3T1602; rT3: catalogue # 38-RT3HR125; DHEA-S: catalogue # 07230105; MP Biomedicals, Burlingame, CA, USA). Total T3 and reverse T3 RIAs were previously validated for northern elephant seals (Ensminger *et al.*, 2014). DHEA-S was validated by demonstrating parallelism of diluted samples to the standard curve (log-logit ANCOVA: *F*_1,5_ = 0.21, *P* = 0.66; observed = 2.34 + 1.02*expected, *r*^2^ = 0.99, *P* < 0.001) and potential for matrix interference effects was tested as described for aldosterone (Beaulieu-McCoy *et al.*, 2017). The calculated hormone recovery was 97 ± 5%. Average intra-assay CVs between sample replicates were 3.2% for tT3, 3.5% for rT3 and 2.3% for DHEA-S.

### Statistical analyses

Statistical analyses were conducted using RStudio statistical software version 1.0.136 (R Core Team, 2016). We used linear mixed models (LMMs), with subject ID as a random effect, to explore hormone variation among samples repeatedly collected from individuals after ACTH administration. Degrees of freedom were estimated using the Kenward–Rogers approximation and *P*-values were determined using the lmerTest package (Kuznetsova *et al.*, 2016); post hoc comparisons were performed using the multcomp package (Torsten *et al.*, 2008).

Responses of each hormone to ACTH administration were assessed with LMMs within Days 1 and 4; if significant differences were detected, we followed with a Dunnett’s post hoc test against the 0-h sample from that day. We evaluated hormone recovery after ACTH administration using only the pre-ACTH (0-h) samples from that day; if differences were detected, we followed with a Dunnett’s test against the Day 1 0-h sample.

We explored associations between hormones using LMMs, with subject ID as random effect. Goodness of fit was calculated with marginal *R*-squared (m*R*^2^) statistics for mixed models (Nakagawa and Schielzeth, 2013) employed in the MuMIn package (Bartoń, 2016).

**Figure 2:**
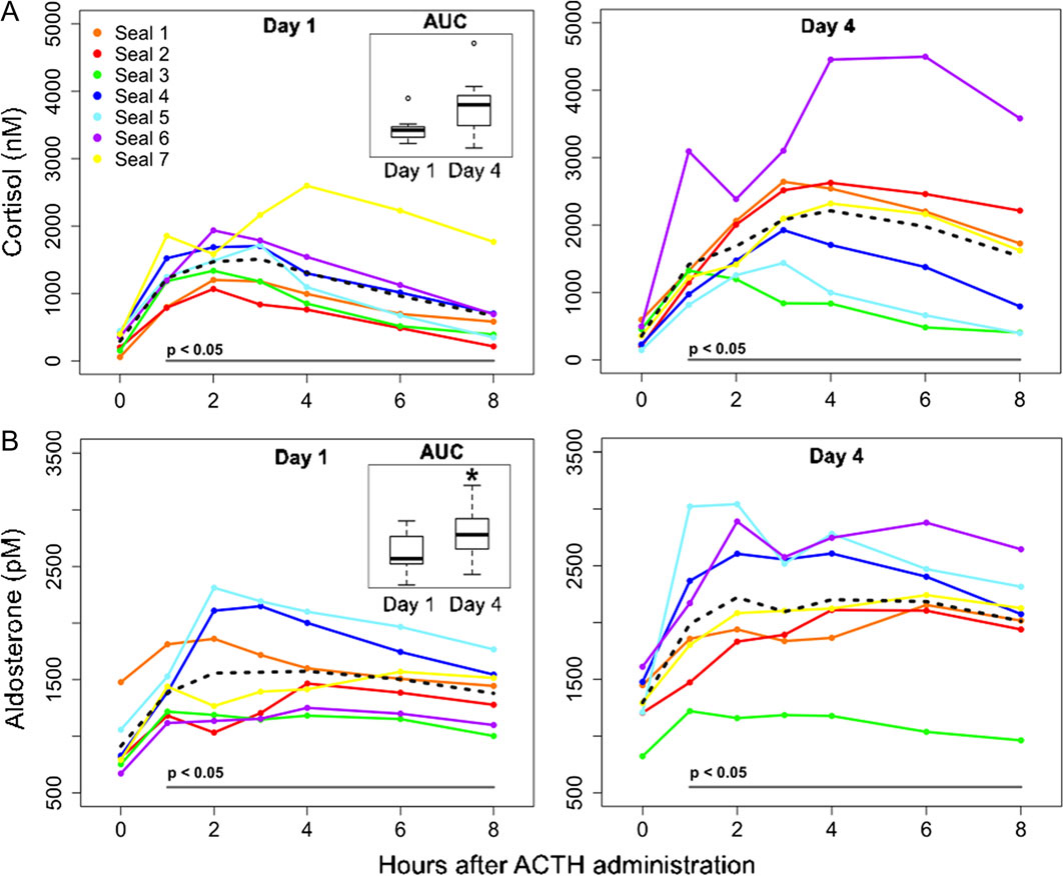
(A) Cortisol concentrations (1–8 h) increased relative to pre-ACTH (0-h) levels following ACTH administration on Days 1 (left panel) and 4 (right panel; LMM: *F*_6, 36_ = 20.7 and 10.1 for Days 1 and 4, respectively, *P* < 0.0001). Total cortisol response (area under the curve, AUC, inset plot) and peak cortisol levels did not differ between Days 1 and 4 (paired *t*-test, AUC: *P* = 0.12, peak: *P* = 0.13). (B) Aldosterone increased relative to pre-ACTH (0-h) concentrations following ACTH administration on Days 1 (left panel) and 4 (right panel; LMM: *F*_6, 36_ = 9.76 and 13.6 for Days 1 and 4, respectively, *P* < 0.0001). Aldosterone concentrations were significantly elevated on Day 4 relative to Day 1 (AUC, inset plot; paired *t*-test, *P* < 0.05). The dashed lines show mean hormone levels. *Denotes statistically significant difference in AUC concentrations between days. The grey bar at the bottom of each panel denotes significant differences in post-ACTH hormone concentrations relative to 0-h levels on each day.

We compared the hormone responses to initial and repeated ACTH administration (Days 1 and 4, respectively) in three ways. (1) For hormones measured in the full set of samples (cortisol and aldosterone), we calculated the total hormone response to ACTH administration on Days 1 and 4 by summing the hormone versus time polygons relative to their initial concentrations and normalized to an 8-h sample duration (Fig. 2) to calculate an integrated area under the curve (AUC) value for each subject. The AUC values for Days 1 and 4 were then compared using paired *t*-tests. We assessed the change in hormone concentration (*∆*) as the change in concentration from the 0-h sample to the peak on that day (0-h subtracted from peak). These values were then compared between Days 1 and 4, using paired *t*-tests, to determine changes in magnitude of response for hormones measured in a subset of samples (e.g. tT3). (2) The maximal (peak) response of each hormone to ACTH was also compared between days using paired *t*-tests. (3) Hormones that were measured in a subset of samples (tT3, rT3 and DHEA-S; each measured in pre-ACTH (0-h) and 4- and 8-h post-ACTH samples) were compared using paired *t*-tests between respective sample times (4-h and 8-h samples on Days 1 and 4). Removal of outliers (e.g. Seal 5) did not have an effect on statistical significance, and data from this animal were retained in the analyses. Due to limited sample size, the effect of sex was not determined in this analysis.

## Results

**Figure 3:**
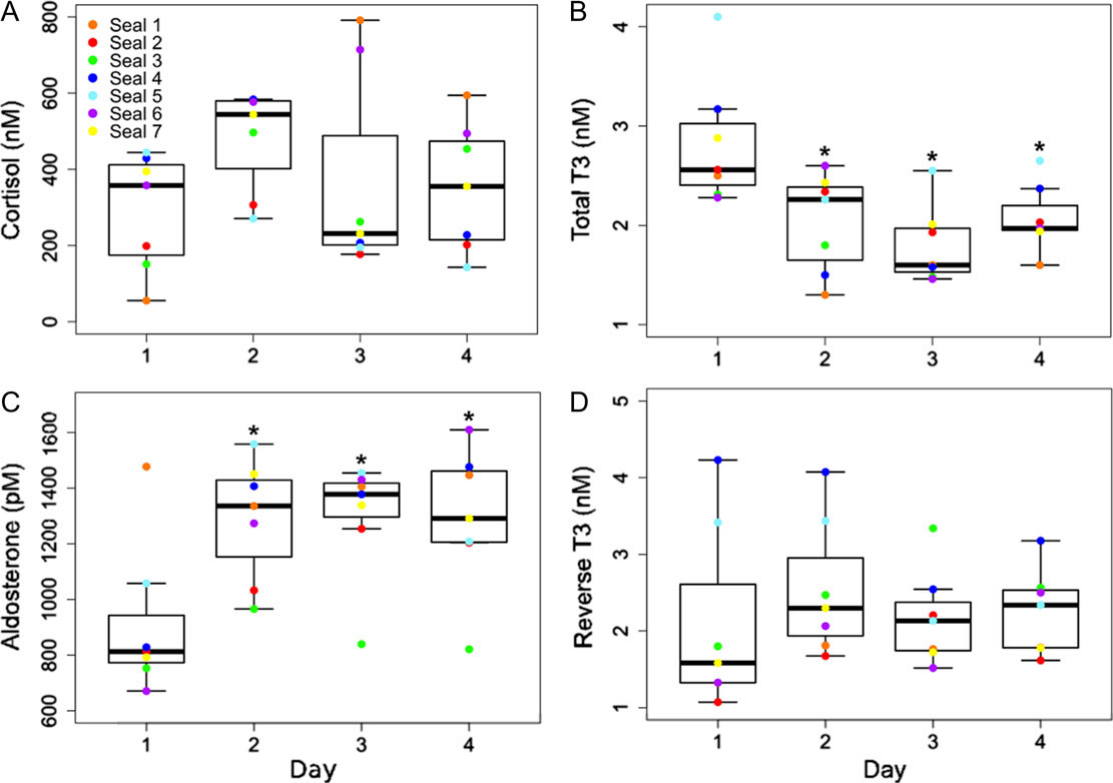
Hormone levels in samples collected prior to ACTH administration (0-h) on each day of the experiment. (A) Cortisol levels recovered to baseline (Day 1 0-h) within 24 h of each ACTH administration. (B) Total T3 0-h concentrations on Days 2–4 were suppressed relative to baseline (LMM: *F*_3, 18_ = 10.5, *P* < 0.0001). (C) Aldosterone 0-h levels on Days 2–4 were elevated relative to baseline (LMM: *F*_3, 18_**=** 8.06, *P* < 0.001). (D) Reverse T3 levels recovered to baseline within 24 h of each ACTH administration. *Denotes significant differences between 0-h hormone levels on Day 1 and subsequent days. Whiskers are within 1.5 standard deviations above and below the third and first quartile, respectively.

ACTH was administered once daily for 4 days to juvenile elephant seals following pre-ACTH (0-h) blood sample collection, and hormone responses to the first and last ACTH administration were evaluated in samples collected 1, 2, 3, 4, 6 and 8 h after injection (Fig. 1) using immunoassays. ACTH administration caused a significant increase in cortisol concentrations relative to 0-h samples on Days 1 and 4 (*Δ*cortisol = 1144 nM on Day 1 and 2047 nM on Day 4; LMM: *F*_6, 36_ = 20.7 and 10.1 for Days 1 and 4, respectively, *P* < 0.0001 for both days). Cortisol levels were significantly elevated in all post-ACTH samples relative to pre-ACTH levels on Days 1 and 4 (Dunnett’s test, *P* < 0.05; Fig. 2A). Cortisol levels in pre-ACTH samples did not vary among Days 1–4 (LMM, *P* = 0.24; Fig. 3A), indicating that cortisol recovered within 24 h of each ACTH administration. There was high variability in the animals’ individual cortisol responses to repeated ACTH administration (Table 1). Some seals exhibited facilitation, or an increase in the magnitude of cortisol secretion in response to the fourth ACTH injection relative to the first (*n* = 4; e.g. Seal 6: *Δ* cortisol = 1578 nM and 4003 nM on Days 1 and 4, respectively). Other animals displayed attenuation, or a decrease in magnitude of the cortisol response (Rich and Romero, 2005) to the fourth ACTH injection relative to the first (*n* = 2; e.g. Seal 3: *Δ* cortisol = 1185 nM and 870 nM on Days 1 and 4, respectively). One animal showed little difference between cortisol responses to ACTH on Days 1 and 4 (Seal 5: *Δ* cortisol = 1275 nM and 1294 nM on Days 1 and 4, respectively). Peak cortisol concentrations and total cortisol secretion in response to ACTH were not significantly different between Days 1 and 4 (paired *t*-test; peak: *P* = 0.13, AUC: *P* = 0.12; Table 1, Fig. 2A).

ACTH administration caused a significant increase in aldosterone concentrations relative to 0-h samples on Days 1 and 4 (*Δ* aldosterone = 383 pM on Day 1 and 708 pM on Day 4; LMM: *F*_6, 36_ = 9.76 and 13.6 for Days 1 and 4, respectively, *P* < 0.0001 for both days). Aldosterone levels were significantly elevated in all post-ACTH samples relative to pre-ACTH levels on Days 1 and 4 (Dunnett’s test, *P* < 0.0001; Fig. 2B). Aldosterone concentrations in 0-h samples from Days 2–4 were significantly elevated relative to the Day 1 0-h sample (LMM: *F*_3, 18_**=** 8.06, *P* < 0.001; Dunnett’s test against Day 1 0-h sample, *P* < 0.001; Fig. 3C). Peak concentrations and total secretion of aldosterone in response to ACTH were significantly higher on Day 4 than on Day 1 (paired *t*-test; peak: *P* < 0.01, AUC: *P* < 0.05; Table 1, Fig. 2B).

**Figure 4:**
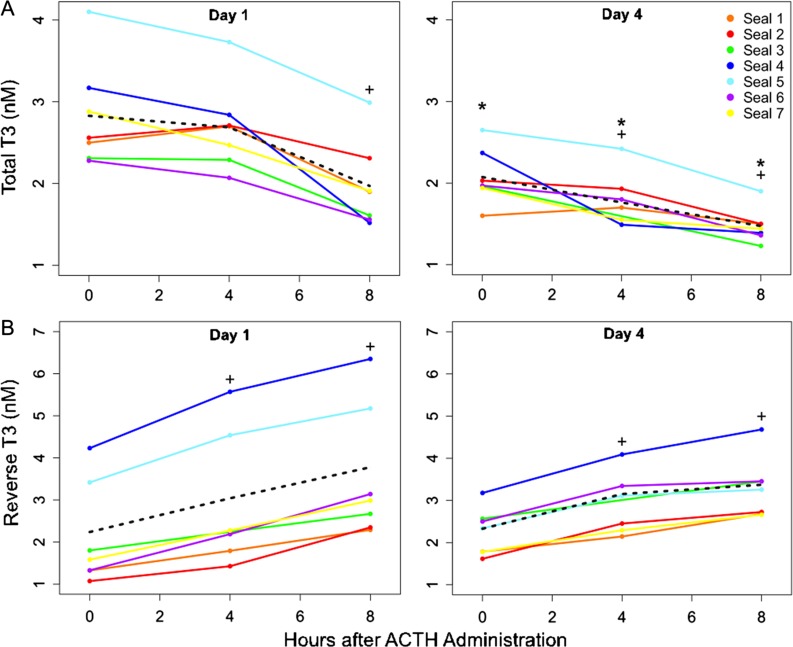
(A) Total T3 concentrations measured 8 h after ACTH administration were significantly decreased relative to pre-ACTH (0-h) levels on Days 1 (left panel) and 4 (right panel). Total T3 concentrations measured post-ACTH were significantly decreased relative to pre-ACTH (0-h) levels on Days 1 (left panel) and 4 (right panel; LMM: *F*_2, 12_ = 25.3, 18.4 for Days 1 and 4, respectively, *P* < 0.0001). Total T3 levels were suppressed on Day 4 relative to Day 1 (paired *t*-test, *P* < 0.001). (B) Reverse T3 concentrations measured 4 and 8 h after ACTH administration were significantly increased relative to pre-ACTH (0-h) levels (LMM: *F*_2, 12_ = 57.0 and 18.4 for Days 1 (left panel) and 4 (right panel), respectively, *P* < 0.0001), but did not differ between days. The dashed lines show mean hormone concentrations. + denotes hormone values that were significantly different (*P* < 0.05) from the 0-h sample on that day. * denotes values that were significantly different (paired *t*-test, *P* < 0.001) between Days 1 and 4.

Thyroid hormones (tT3 and rT3) and DHEA-S concentrations were measured in 0-h samples from Days 1–4 and in samples collected 4 and 8 h after ACTH administration on Days 1 and 4. ACTH caused a significant decrease in tT3 levels on both Days 1 and 4 (LMM: *F*_2, 12_ = 25.3, 18.4 for Days 1 and 4, respectively, *P* < 0.0001; Fig. 4A). On Day 1, tT3 concentrations were significantly decreased in the 8-h sample (Dunnett’s test, *P* < 0.001) compared with pre-ACTH levels, but not in the 4-h sample (*P* = 0.44). On Day 4, tT3 was significantly decreased related to pre-ACTH levels in both 4-h and 8-h post-ACTH samples (Dunnett’s test, *P* < 0.001). The magnitude of tT3 responses to ACTH was not different between Days 1 and 4 (paired *t*-test, *P* = 0.08). Total T3 levels in 0-h samples from Days 2 to Day 4 were significantly lower than the 0-h sample from Day 1 (LMM: *F*_3, 18_ = 10.5, *P* < 0.0001; Fig 3B), and were further suppressed in post-ACTH samples (4-h and 8-h) from Day 4 relative to those from Day 1 (paired *t*-test, *P* < 0.001).

**Figure 5:**
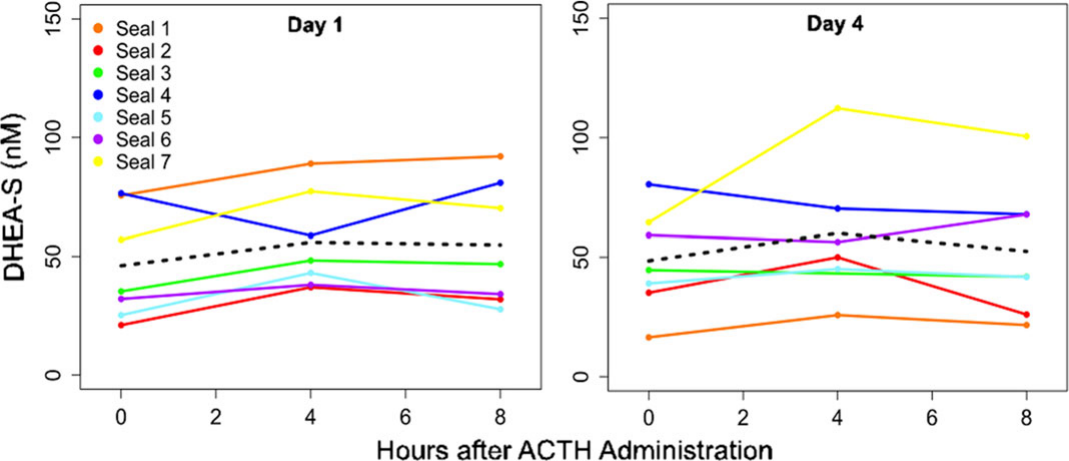
DHEA-S concentrations did not change following ACTH administrations on Day 1 (left panel; *P* > 0.05) or Day 4 (right panel; *P* > 0.05). The dashed lines show mean hormone concentrations.

Reverse T3 levels were significantly increased 4 h and 8 h after ACTH administration relative to 0-h concentrations on Days 1 and 4 (LMM: *F*_2, 12_ = 57.0 and 18.4 for Days 1 and 4, respectively, *P* < 0.0001; Dunnett’s test against the 0-h for each day, *P* < 0.001; paired *t*-test, *P* < 0.05; Fig. 4B). However, rT3 concentrations did not vary between 0-h samples on Days 1–4 (LMM, *P* = 0.95; Fig. 3D). There were no significant differences in post-ACTH rT3 concentrations on Days 1 and 4 (paired *t*-test, *P* = 0.76 and *P* = 0.50 for Days 1 and 4, respectively). DHEA-S concentrations were not affected by ACTH (LMM, *P* = 0.06; Fig. 5) and did not differ between Days 1 and 4 (paired *t*-test, *P* = 0.31).

**Figure 6:**
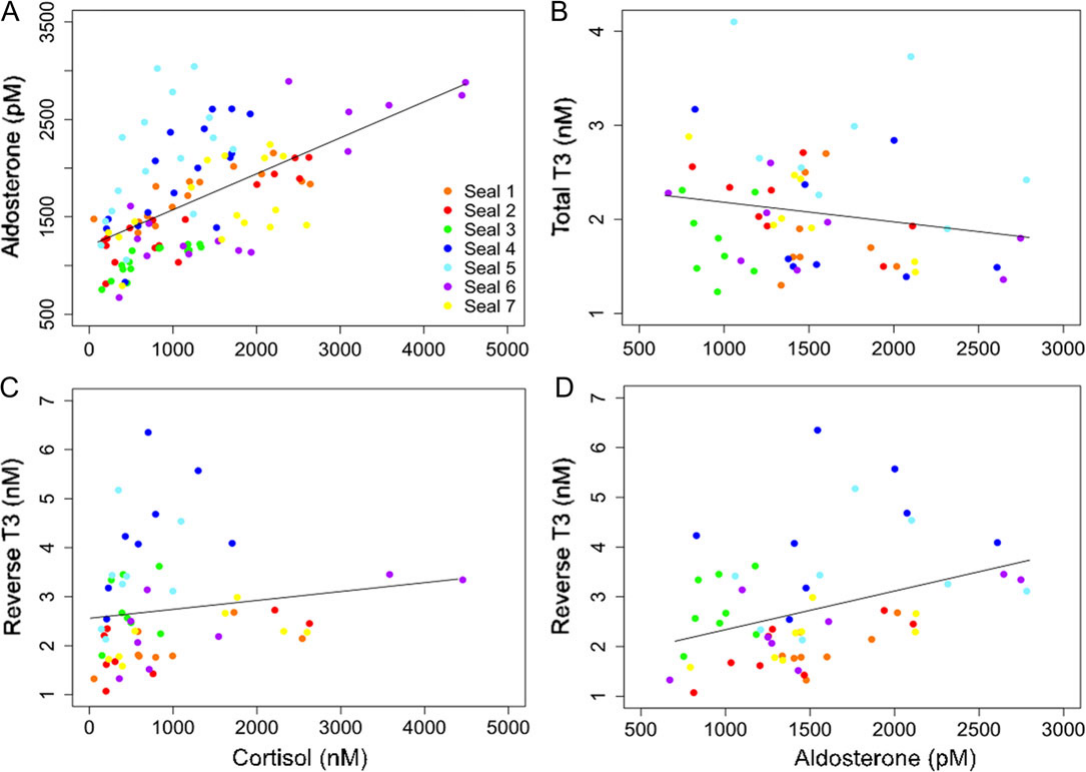
Associations between hormone levels in samples collected during the experiment. (A) Cortisol and aldosterone were positively associated (LMM: *F*_1, 106.8_ = 105, *P* < 0.0001; m*R*^2^ = 0.38). (B) Aldosterone and total T3 were negatively associated (LMM: *F*_1, 53.5_ = 8.26, *P* < 0.001; m*R*^2^ = 0.12). (C) Cortisol and reverse T3 were positively associated (LMM: *F*_1, 7.47_ = 6.38, *P* < 0.05; m*R*^2^ = 0.29). (D) Aldosterone and reverse T3 were positively associated (LMM: *F*_1, 7.66_ = 5.88, *P* < 0.05; m*R*^2^ = 0.26).

Cortisol was positively associated with aldosterone (LMM: *F*_1, 107_ = 105, *P* < 0.0001; m*R*^2^ = 0.38; Fig. 6A) and rT3 (LMM: *F*_1, 49_ = 12.0, *P* < 0.001; m*R*^2^ = 0.09; Fig. 6C). Aldosterone was negatively associated with tT3 (LMM: *F*_1, 54_ = 8.26, *P* < 0.001; m*R*^2^ = 0.12; Fig. 6B) and positively associated with rT3 (LMM: *F*_1, 50_ = 5.88, *P* < 0.001; m*R*^2^ = 0.10; Fig. 6D).

## Discussion

We evaluated the endocrine responses of free-ranging marine mammals to repeated HPA axis activation. We administered ACTH once daily for four consecutive days to juvenile northern elephant seals to simulate the physiological responses that a marine mammal may experience to frequently occurring natural or anthropogenic stressors. We measured corticosteroid and thyroid hormone levels in samples collected prior to each ACTH administration and for 8 h following the first and fourth administration. Elephant seals in this study showed similar magnitudes of cortisol release on Day 4 as on Day 1, suggesting that they maintained adrenal sensitivity to repeated ACTH stimulation for at least 4 days. Aldosterone and tT3, however, showed different responses to the initial and repeated ACTH administration. The magnitude of the aldosterone response increased following repeated ACTH administrations. Total T3 concentrations decreased following multiple ACTH administrations. These differential responses to single and repeated ACTH administrations suggest that aldosterone and tT3 may be useful markers to aid in distinguishing between acute and repeated stress states.

Cortisol significantly increased in response to ACTH on Days 1 and 4 of the study. While cortisol release was much higher than has been previously reported for other marine mammals in response to exogenous ACTH (e.g. harbour seals and Steller sea lions, Mashburn and Atkinson, 2008; Keogh and Atkinson, 2015), values were within range of GC concentrations previously reported in juvenile northern elephant seals (Champagne *et al.*, 2015; Khudyakov *et al.*, 2015, 2017). ACTH response samples, however, were only collected 2 h after administration in the aforementioned studies. In this study, we collected samples for 8 h following ACTH administration to more fully capture the endocrine response.

The magnitude of cortisol responses did not vary between single and repeated ACTH administration, as the AUC and peak values were not statistically different between Days 1 and 4. However, all animals had elevated cortisol concentrations for 8 h following ACTH administration on Days 1 and 4. While post-ACTH response samples were not collected on Days 2 and 3 of this study, we conducted pilot trials that demonstrated that similar responses are expected (data not shown). This likely resulted in a substantial endocrine alterations that may simulate a chronically stressed state. Despite this, all animals retained a high level of adrenal responsiveness to ACTH, and cortisol levels returned to baseline within 24 h of each ACTH dose in all seven study animals. This response suggests that juvenile northern elephant seals retain the capacity to repeatedly mount responses to ACTH administration for at least 4 days.

The wide variation in responses among individual subjects suggests that physiological differences between specific animals account for the variable cortisol responses to repeated ACTH administration. While the highest responses on Days 1 and 4 were in females (Seal 7 and Seal 6), the two females were slightly smaller than the males, and therefore received a higher mass-specific dose of ACTH. This was coincidental as the mass of juvenile elephant seals does not generally vary by sex (Crocker *et al.*, 2012; Jelincic *et al.*, 2017). Previous studies have shown no sex differences in baseline cortisol, aldosterone or ACTH concentrations in juvenile northern elephant seals (Kelso *et al.*, 2012; Jelincic *et al.*, 2017). There was also no effect of sex on *Δ* cortisol measured 2 h after one ACTH administration in juveniles from this and our other studies (10 females, 10 males, *P* > 0.05; Khudyakov *et al.*, 2015, 2017). Variability in individual GC responses to exogenous ACTH or other types of stressors (e.g. handling, capture, etc.) has been observed in other marine mammals (e.g. Steller sea lions, Mashburn and Atkinson, 2008), fish and terrestrial species (Bry, 1982; Guimont and Wynne-Edwards, 2006; Cockrem, 2013), which suggests that GC concentrations are highly variable across species and stressors and may therefore be less reliable indicators of specific stress states (Dickens and Romero, 2013).

Aldosterone was significantly elevated in response to ACTH administration both on Days 1 and 4. While aldosterone levels measured in this study were slightly higher than those reported in harbour seals in response to ACTH (Keogh and Atkinson, 2015), they were within range of values previously reported for juvenile northern elephant seals (Champagne *et al.*, 2015; Khudyakov *et al.*, 2015, 2017). Repeated ACTH administration significantly affected aldosterone secretion: peak and AUC values of aldosterone were higher after the fourth ACTH administration (Day 4) relative to those measured after the first (Day 1). All animals showed elevated aldosterone concentrations for at least 7 h (and likely longer) following ACTH administration on Days 1 and 4. Unlike cortisol, aldosterone concentrations did not recover to baseline values within 24 h of the first ACTH administration and remained elevated in 0-h samples collected on Days 2, 3 and 4. Therefore, it is likely that aldosterone remained elevated throughout the duration of the experiment.

All but one seal (Seal 3), exhibited facilitation of aldosterone secretion on Day 4 compared with Day 1, including individuals that had displayed attenuation or no change in their cortisol responses to multiple ACTH administrations (Fig. 2). These data suggest that aldosterone is sensitive to repeated HPA axis activation, and may therefore serve as an informative indicator of repeated stress states in marine mammals.

Significant increases in aldosterone levels in response to acute stressors (e.g. ACTH administration, cold water, veterinary examination) have been observed in multiple marine mammal species, including pinnipeds such as northern elephant seals (Ensminger *et al.*, 2014; Champagne *et al.*, 2015) and Pacific harbour seals (Gulland *et al.*, 1999; Keogh and Atkinson, 2015), and cetaceans, such as Atlantic bottlenose dolphins (Houser *et al.*, 2011; Ortiz and Worthy, 2000b; St. Aubin *et al.*, 1996) and spotted dolphins (St Aubin *et al.*, 2013). The ability to regulate aldosterone concentrations in the contexts of normal and stress physiology may be especially essential for marine mammals that ingest large amounts of salt and water during foraging and feeding and that must maintain appropriate osmoregulatory function while diving (Atkinson *et al.*, 2015; Ortiz *et al.*, 2000a; St Aubin, 2001). In terrestrial animals, exposure to psychosocial and cold stress has been shown to directly increase renin production, which is likely mediated by the sympathetic nervous system (Cassis *et al.*, 1998; Clamage *et al.*, 1976; Groeschel and Braam, 2011). Angiotensin II has also been shown to increase in response to stress in rats, directly increasing aldosterone levels by activating Type 1 angiotensin II receptors in the adrenal cortex (Saavedra and Benicky, 2007). Therefore, it is possible that aldosterone secretion in response to stress in marine mammals occurs indirectly, via stress-induced sympathetic activation of the renin–angiotensin–aldosterone system (Houser *et al.*, 2011). However, the positive correlation between cortisol and aldosterone concentrations and responsiveness to ACTH reported in this and other marine mammal studies using anaesthetized animals (with minimal sympathetic activation in response to research handling), suggests that the HPA axis may play a major role in aldosterone regulation in response to stress (Champagne *et al.*, 2015).

ACTH administration had no effect on DHEA in this study. Significant increases in DHEA have been reported in humans, non-human primates, and cattle in response to repeated stress, suggesting that this hormone precursor may be used to buffer effects of sustained elevation of GCs (Lennartsson *et al.*, 2012; Maninger *et al.*, 2010; Marinelli *et al.*, 2007). However, experiments using white-crowned sparrows have shown that DHEA is either unaffected or suppressed in response to acute stress (Krause *et al.*, 2014), and other species such as bullfrogs (*Rana catesbeiana*), lizards (*Lacerta viridis*), grass snakes (*Natrix natrix*) and ducks display low levels of CYP17A1 enzyme activity relative to mammals (Phillips *et al.*, 1962; Sandor *et al.*, 1963; Ulick and Solomon, 1960). Therefore, adrenal synthesis of DHEA in these species, and potentially marine mammals as well, may be maximal at baseline and adrenal stimulation in response to stress may have no effect on production due to low CYP17A1 expression or activity.

ACTH administration had a significant impact on thyroid hormone levels. ACTH significantly suppressed tT3 levels and increased rT3 on both Days 1 and 4. The highest total T3 values in our study were ~2-fold higher than tT3 values described for adult male northern elephant seals, even after suppression by ACTH (Ensminger *et al.*, 2014). Reverse T3 values reported here were almost 50-fold higher than those measured in adult males, but within the range of values previously reported for juvenile northern elephant seals in response to exogenous ACTH (Champagne *et al.*, 2015; Ensminger *et al.*, 2014). Total T3 levels decreased within 8 h of the first ACTH administration and remained suppressed relative to the baseline (Day 1 0-h) sample for the remainder of the experiment. However, while the first ACTH dose significantly decreased tT3 production and subsequent doses caused further suppression, the magnitude of tT3 suppression on Days 1 and 4 was not different. These results are not unexpected as cortisol has been shown to suppress conversion of T4 to the biologically active T3 and promote its conversion to the biologically inactive rT3 via its effects on deiodinase enzymes (Bianco *et al.*, 2006, 1987). Accordingly, levels of rT3 increased within 4 h of ACTH administration, which is consistent with other studies in this species (Champagne *et al.*, 2015). Unlike tT3, however, there was no difference in rT3 responses to the first and fourth ACTH administration. This may be due to differential regulation of deiodinase enzymes (Mullur *et al.*, 2014; Ortiga‐Carvalho *et al.*, 2016) or hormone clearance rates by the liver, as rT3 is unbound by carrier proteins and therefore may be cleared more rapidly than tT3 (Chopra, 1976). Reverse T3 and cortisol levels, however, were positively associated in response to a single ACTH administration in juveniles and adult male elephant seals sampled early in the breeding season (Champagne *et al.*, 2015; Ensminger *et al.*, 2014). This suggests that thyroid sensitivity to HPA axis activation is dependent on the number of ACTH administrations (single vs. repeated) and life-history stage in elephant seals, and that repeated stress has a significant inhibitory effect on thyroid hormone production.

Associations between HPA and HPT axes have been shown in laboratory rodents (Helmreich *et al.*, 2005) and marine mammal species including belugas, in which capture and handling stress resulted in suppression of T3 (St. Aubin and Geraci, 1988). The inhibitory effect of stress on T3 has significant implications as thyroid hormones regulate basal metabolic rate via effects on mitochondrial proliferation and lipid and protein metabolism (Atkinson *et al.*, 2015; Fair *et al.*, 2011). Stress-induced disruption in thyroid hormone production can therefore greatly impact health and fitness in species that fast during critical life-history stages (e.g. breeding, molting, migration). For example, mean thyroid hormone levels do not change in male northern elephant seals over their extended breeding fast, enabling them to maintain elevated metabolic rates for its duration. Furthermore, the ability of individual seals to elevate T3 is associated with higher daily energy expenditure and breeding success (Crocker *et al.*, 2012; Lee *et al.*, 2017). Metabolic suppression, even for short periods during this life-history stage, could lead to an inability to sustain reproductive behaviour and decrease fitness.

This study demonstrates the importance of measuring multiple endocrine variables in addition to GCs in response to repeated ACTH administration. We demonstrated that cortisol responses to HPA axis stimulation varied between individuals but not by the number of ACTH administrations. GC responses to stress are therefore likely to be more significantly influenced by physiological variability between animals than aldosterone and tT3, which displayed consistent differences between their responses to single and multiple ACTH administrations. Sensitization of the aldosterone response and suppression of tT3 by repeated ACTH administration suggests mechanisms by which repeated stress may affect marine mammal health and fitness (i.e. altering osmoregulation or cardiovascular adjustments and suppressing metabolism). Aldosterone and tT3 also demonstrate potential as markers of long-term stress, such as those that would be elicited by repeated anthropogenic disturbance, given contextual information about their natural life-history variation. These data suggest that aldosterone and thyroid hormone levels may be informative biomarkers of repeated stress in marine mammals. Determining the range and variability in baseline concentrations will help to further contextualize the response of HPA and HPT axes to chronic stress in elephant seals and other marine mammals (Jelincic *et al.*, 2017). Characterizing the downstream consequences of stress, including alterations in gene expression and metabolic pathways, will offer additional insights for differentiating between physiological responses to acute and repeated stress.

Anthropogenic activity is likely to continue to increase in marine ecosystems, increasing the likelihood of human interactions with marine life that may elicit stress and compound the physiological extremes already experienced by these animals as a consequence of ocean living (Maxwell *et al.*, 2013). Geographical ranges of many marine mammals, including threatened species, overlap with areas associated with high human activity like fishing, shipping, or military sonar use (Davidson *et al.*, 2012; Falcone *et al.*, 2017; Schorr *et al.*, 2014). Therefore, the ability to accurately measure homeostatic loads in marine mammals exposed to anthropogenic disturbance and predict their effects on fitness is increasingly important for conservation practitioners working to protect marine ecosystems and their top predators (Fair and Becker, 2000).

## Supplementary Material

Supplementary Data
