# Supplementary material for: Repeated adrenocorticotropic hormone administration alters adrenal and thyroid hormones in free-ranging elephant seals
Source: Conserv Physiol. 2018 Jul 17;6(1):coy040. doi: 10.1093/conphys/coy040 (PMC6048993; doi:10.1093/conphys/coy040)
Supplement: Supplementary Data [file coy040_supplementary_materials.docx]

**Figure S1**

**Figure S1.** The enzyme-linked immunosorbent assay (ELISA) used to measure aldosterone was validated in part by showing parallelism of serially diluted pooled seal serum (with high concentrations of endogenous aldosterone collected during this study) to the standard curve (A) and plotting observed versus expected values of the serial dilution (B). A) The standard curve is shown in black while the serially diluted serum is shown in blue (adjusted by +0.2 on the y-axis for clarity; numeric values show the fractional dilution of the serum). Parallelism was assessed from the linear region of the standard curve, shown in the grey rectangle, using an ANCOVA of the absorbance values against aldosterone concentration and sample type (kit standard or diluted serum; full model ANCOVA: F_1, 5_ = 643, p < 0.0001). The interaction term (aldosterone concentration * sample type) was not significant (p = 0.1) suggesting that the slopes do not significantly differ between the standards and serially diluted samples. B) Using a second pool of seal serum, we assessed the assay by evaluating observed values against those expected based on the degree of serum dilution (95% confidence interval shown in blue). There was a significant relationship between the observed and expected values (observed = 506 + 0.79*expected; r^2^ = 0.98, F_1, 3_ = 197, p < 0.001).
